# Mediterranean Diet and Oxidative Balance During Pregnancy: Molecular Insights into Mitigating the Impact of Environmental Pollution

**DOI:** 10.3390/cimb48010115

**Published:** 2026-01-21

**Authors:** Eirini Kontopidou, Areti Kourti, Apostolos Athanasiadis, Aikaterini Itziou

**Affiliations:** 1 Department of Midwifery, School of Health Sciences, University of Western Macedonia, 50200 Ptolemaida, Greece; 2 Laboratory of Biochemistry, School of Medicine, AHEPA University Hospital, Aristotle University of Thessaloniki, 54636 Thessaloniki, Greece; aretikourti@auth.gr; 3 Third Department of Obstetrics and Gynaecology, School of Medicine, Faculty of Health Sciences, Aristotle University of Thessaloniki, 54636 Thessaloniki, Greece; apathana@auth.gr

**Keywords:** Mediterranean diet, pregnancy, oxidative stress, antioxidant, pollution

## Abstract

Pregnancy represents a period of heightened oxidative demand in which maternal metabolic adaptations are tightly regulated by redox-sensitive molecular pathways. Imbalances in these systems have been associated with gestational complications, impaired placental function, and long-term effects on offspring health. This review examines the molecular mechanisms through which adherence to the Mediterranean diet (MD) influences oxidative balance during pregnancy. We summarize evidence on how MD-derived bioactives regulate oxidative stress pathways and affect oxidative stress biomarkers, as well as the expression of antioxidant enzymes such as superoxide dismutase and glutathione peroxidase. At the same time, certain MD foods containing environmental contaminants may potentially attenuate its protective effects. In addition, the review explores molecular insights into how the MD may counteract oxidative stress induced by environmental pollutants through modulation of redox signaling and detoxification pathways. By integrating biochemical, molecular, and environmental perspectives, this review highlights the MD as a potential nutrigenomic intervention to optimize oxidative balance, support healthy pregnancy outcomes linked to environmental pollution.

## 1. Introduction

### 1.1. Pregnancy as a State of Heightened Oxidative Demand

#### 1.1.1. Physiological Changes in Oxygen Consumption and Mitochondrial Activity

During pregnancy, both the developing fetus and the mother change their metabolism, regulate oxygen consumption (VO_2_), and mitochondrial activity in order to maintain redox balance, support placental development, and buffer against oxidative damage associated with increased metabolic activity [1]. Evidence from human physiological studies and placental tissue analyses consistently demonstrates how mitochondrial function changes during normal pregnancy and how the impairment of mitochondrial regulatory control may contribute to pregnancy disorders [2,3,4]. At the cellular level, mitochondria adapt by regulating respiratory capacity, adenosine triphosphate (ATP) production, and substrate utilization for energy production [5,6]. Sánchez-Aranguren et al. [3] describe these adaptations as a finely regulated increase in oxidative phosphorylation (OXPHOS) activity, supported by control mechanisms that prevent excessive electron escape and maintain redox homeostasis. The placenta, which is one of the most active organs during pregnancy, shows this bioenergetic flexibility. Human placental studies provide stronger translational relevance than animal models, showing structural remodeling of placental mitochondria such as changes in their cristae structure, enzyme expression, and mitochondrial fusion and fission processes. These changes help to meet the growing energy needs while the production of reactive oxygen species (ROS) is low [6,7]. However, insight into mitochondrial dynamics still derives from animal or in vitro trophoblast models, which may not fully capture the complexity of human pregnancy. Although these mechanisms are adapted to pregnancy period, oxidative stress is increased due to the higher VO_2_ and the demand on mitochondria. Observational studies consistently report elevations in oxidative stress biomarkers across gestation, counterbalanced by upregulation of antioxidant systems, including glutathione-dependent pathways and enzymatic scavengers [8]. However, when these mechanisms do not work properly, they can disrupt mitochondrial homeostasis and cause oxidative damage and disrupt cellular signaling. Findings from systematic reviews by Ibrahim et al. [9] illustrate that oxidative stress biomarkers increase during pregnancy and often result to negative outcomes. Nevertheless, most evidence in this area is associative, limiting causal inference.

Moreover, mitochondria function as key regulators of inflammatory and metabolic signals. In the uterine vascular system, as oxygen consumption increases, it leads to impaired susceptibility of mitochondrial pathways to inflammatory signals. Human studies by Mandalà et al. [10] support that uterine-vascular adaptation is formed by oxidative and inflammatory signals. This regulates endothelial nitric oxide bioavailability and vascular remodeling, which are essentials for uteroplacental blood flow. These functions can be compromised by dysregulated mitochondrial ROS and lead to an increased risk for gestational hypertension and preeclampsia, although whether mitochondrial dysfunction is a primary factor or a secondary consequence of vascular pathology remains uncertain. Recent studies highlight that mitochondrial activity and systemic oxidative stress are associated with maternal cellular aging markers. An observational cohort study by Etzel et al. [11] reports that oxidative stress, systemic inflammation, and maternal telomere length are linked, suggesting that higher metabolic stress may lead to increased telomere shortening. Although the causal direction remains unresolved, the findings underscore the systemic effects of mitochondrial redox imbalance across gestation. Toledano et al. [4] suggest that altered mitochondrial homeostasis, in which reduced OXPHOS efficiency appears, higher ROS production, and impaired mitophagic processes, leads to pathophysiological pathways in preeclampsia, intrauterine growth restriction (IUGR), and gestational diabetes. When these processes occur, abnormal placental oxygenation patterns and reduced mitochondrial resilience are developing [12]. Moreover, Sánchez-Aranguren et al. [3] emphasize that early pregnancy is a particularly vulnerable period, as inadequate mitochondrial adaptation during placentation can set the stage for important persistent oxidative stress and vascular dysfunction.

Overall, the literature concludes that physiological increases in oxygen consumption and mitochondrial activation are important for a healthy pregnancy. On the other hand, these mechanisms must not exceed to prevent oxidative stress and inflammation. Human placental studies provide robust evidence for adaptive mitochondrial remodeling, while insights largely rely on animal and in vitro models. The key appears to be the balance between increased energy production and controlled ROS production for healthy placental development, vascular remodeling, and maternal systemic health. Researchers keep searching in order to clearly understand biomarker profiles and pathways, trying to interpret mitochondrial function for early detection and therapeutic intervention in pregnancy-related complications. Future research should prioritize longitudinal human studies integrating functional mitochondrial biomarkers with clinical outcomes.

#### 1.1.2. Redox-Sensitive Regulation of Placental Development and Fetal Growth

In the first trimester of pregnancy, physiologically, oxygen levels are low, which contributes to trophoblast invasion and villous development. In this redox-sensitive environment, the controlled production of ROS is a key factor that influences gene expression, mitochondrial activity, and angiogenic factor regulation. Experimental and human placental studies demonstrate that these redox signals affect pathways involving transcription factors such as Hypoxia-Inducible Factor 1-Alpha (HIF-1α), Nuclear factor erythroid 2-related factor 2 (NRF2), and Nuclear Factor kappa-light-chain-enhancer of activated B cells (NF-κB), which are essentials for placental morphogenesis [13]. In the second trimester of pregnancy, the increase in oxygen availability demands a reset of redox homeostasis. This happens to allow normal vascular expansion and nutrient transport.

Placental development is adversely affected, when redox balance is disrupted. Excessive ROS generation, often resulting from mitochondrial dysfunction or inflammatory activation, can impair the viability of trophoblast, change communication from cell to cell, and disrupt vascular remodeling. Human observational studies and placental histopathology analyses by Sultana et al. [14] highlight that oxidative stress affects uteroplacental blood flow by promoting endothelial dysfunction and compromising spiral artery transformation, changes that reduce oxygen and nutrient delivery to the fetus. In addition to the above, Zhang et al. [15] supports that oxidative stress at the maternal–fetal interface makes vascular maladaptive in reproductive system disorders, since redox imbalance is linked to placental pathology. While animal models provide experimental support, human data remain largely correlative.

Recent research supports the idea that oxidative stress relates to negative fetal outcomes, particularly fetal growth restriction (FGR). Systematic analysis about pregnancies affected by FGR found that oxidative stress biomarkers increase, especially the lipid peroxidation products and there are reductions in antioxidant enzymes [16]. These findings support the view that oxidative stress is not only a consequence of impaired fetal development but a central mechanism of growth-limiting placental insufficiency. Moreover, oxidative stress causes changes across gestation in cases of intrauterine growth restriction, with early disruptions which can lead to later placental structure and functional deterioration [17].

Maternal metabolic status can also influence placental redox regulation. Another factor which increases systemic oxidative stress and inflammatory burden and alters placental cytokine expression and antioxidant capacity is obesity. All the above changes influence the efficiency of placenta and are linked with reduced fetal growth patterns [18]. Maternal physiology, placental function, and fetal development are connected through redox-dependent pathways, as the previous findings underscore. These associations are supported primarily by observational data, underscoring the need for interventional studies.

At the molecular level, redox-sensitive regulation is very important in processes that support fetal growth. AMP-activated protein kinase (AMPK) and Mammalian target of rapamycin (Mtor) pathways are influenced by ROS and regulate cellular energy sensing, protein synthesis, and mitochondrial biogenesis. Additionally, redox signals shape placental capacity. Redox regulation affects nutrient transporter expression directly impacts the availability of glucose and amino acids to the fetus. According to Divella et al. [19], persistent redox imbalance during critical periods of development may generate long-term effects. This will increase the risk of cardiometabolic diseases in offspring later in life. Developmental evidence is compelling but largely indirect, relying on long-term cohort associations rather than direct human data.

Current research indicates that vulnerable to redox-dependent regulation is both placental development and fetal growth. Human observational and placental studies provide strong associative evidence, complemented by animal data. While physiological ROS levels are very important for signaling, excessive oxidative stress can disrupt trophoblast function, vascular development, and nutrient transport, ultimately compromising fetal growth. New targeted therapeutic strategies will occur if a better understanding of redox thresholds, temporal patterns, and interacting maternal factors can be achieved. Interventions such as antioxidant therapies, modulation of mitochondrial function, and redox-targeted pharmacological agents offer promising solutions for restoring placental homeostasis and improve pregnancy outcomes.

### 1.2. Oxidative Imbalance and Pregnancy Complications

During normal gestation, oxygen consumption and mitochondrial activity progressively increase, due to escalating metabolic demands in the placenta. Longitudinal human studies confirm that this process is accompanied by a compensatory enhancement of antioxidant defense mechanisms that preserve redox equilibrium [20]. When placenta develops early, under low oxygen conditions, embryonic tissues are protected from oxidative injury, while when placenta develops late, oxygen tension, which supports placenta, is increased. Moreover, when this physiological oxygen transition fails, including exposure to oxygen concentrations levels, higher than normal, adverse obstetric, and neonatal outcomes arise, indicating how disrupted redox homeostasis occurs very easily in pregnancy [21].

Complicated pregnancies have increased risk of vascular dysfunction due to oxidative stress. Excess ROS decreases the bioavailability of nitric oxide. This occurs through direct scavenging of reactive species and by disrupting the function of endothelial nitric oxide synthase, which reduces vasodilation and increases vascular resistance. Additionally, oxidative stress promotes endothelial inflammation, leukocyte adhesion, and pro-thrombotic states, reinforcing the multisystem nature of preeclampsia, as well supported by human vascular studies and placental analyses [22].

Preeclampsia is one of the most extensively studied pregnancy disorders that is linked to oxidative stress. There are two types of preeclampsia, Type I (placental) and Type II (maternal), that have differences in disease onset and progression [23]. In Type I preeclampsia abnormal placentation is met, characterized by insufficient trophoblast invasion and improper remodeling of the spiral arteries. These defects subject the placenta to repeated cycles of hypoxia and reoxygenation, resulting in excessive ROS generation and oxidative injury. In contrast, Type II preeclampsia is more strongly associated with pre-existing maternal cardiovascular or metabolic dysfunction, where oxidative stress further heightened endothelial susceptibility even in the presence of relatively preserved placentation. While this framework is useful, overlap between phenotypes remains substantial.

At the molecular level, oxidative stress activates redox-sensitive signaling pathways, including NF-κB and mitogen-activated protein kinases, promoting inflammation, apoptosis, and anti-angiogenic signaling observed in human studies [24,25]. An increased oxidative load stimulates placental secretion of anti-angiogenic factors such as soluble fms-like tyrosine kinase-1 (sFlt-1), which counteracts vascular endothelial growth factor (VEGF) and placental growth factor (PlGF), leading to systemic endothelial dysfunction. The resulting vascular imbalance presents as hypertension, proteinuria, and multi-organ involvement [26].

Beyond hypertensive disorders, oxidative stress contributes significantly to metabolic complications of pregnancy. Gestational diabetes mellitus (GDM) is characterized by hyperglycemia-induced ROS generation, mitochondrial dysfunction, and impaired insulin signaling within placental and maternal tissues [27]. Placental mitochondria in GDM shows increased fusion activity, which is a response to metabolic efficiency and redox stability [28]. Nevertheless, this is not enough, and oxidative stress persists and disrupts placental nutrient transport and vascular function.

FGR is related to oxidative imbalance. In such pregnancies, different expressions of mitochondrial and glycolysis-regulatory genes have been observed, highlighting increased placental bioenergetic capacity and inefficient substrate use [29]. These changes reduce the availability of energy to the fetus, which can lead to long-term developmental consequences.

Exogenous oxidative stress factors increase vulnerability of pregnancy. Maternal infections, including COVID-19, negatively contribute to this process. Endothelial function and placental blood circulation is adversely affecting and inflammation such as oxidative stress are deteriorating [30]. Lifestyle factors and environmental exposures similarly increase oxidative load, increasing the risk of preeclampsia, GDM, preterm birth, and adverse neonatal outcomes [31]. While these associations are robust, disentangling direct oxidative effects from confounding socioeconomic and health factors remains challenging.

In conclusion, evidence supports that oxidative stress is a common reason for various pregnancy complications [32]. Unfortunately, antioxidant therapies have produced inconsistent clinical outcomes, likely due to variations in diseases and timing of intervention. It is important to gain a better understanding of redox-sensitive pathways to develop targeted biomarkers and precision-based prevention strategies.

### 1.3. Maternal Diet and Environmental Exposures as Modulators of Redox Homeostasis

Critical factors of redox homeostasis during pregnancy are maternal diet and environmental exposures because they influence maternal metabolic adaptation, placental function, and fetal development [31].

Nutritional status plays a central role in regulating oxidative balance and influencing mitochondrial function, substrate metabolism, and antioxidant defense capacity. When diet is enriched with lot of saturated fats, carbohydrates which are refined, and processed foods with high calories, there is increased ROS generation and mitochondrial inefficiency while there is limiting antioxidant buffering at the same time. In contrast, dietary plans rich in antioxidants, unsaturated fatty acids, and essential micronutrients help redox to be stable and support metabolic flexibility. Diamanti-Kandarakis et al. [33] emphasize that nutrition can act as a crucial factor of oxidative stress in reproductive and metabolic disorders and can influence endocrine signaling, insulin sensitivity, and inflammatory pathways relevant to pregnancy.

The impact of maternal diet on oxidative status attracted epidemiological research. Data from the NELA Birth Cohort demonstrate that during pregnancy healthier dietary patterns can reduce biomarkers of oxidative stress in mothers and oxidative stress in offspring. This supports that if there is a balance in maternal redox, this can have long-term consequences to the future generations [34]. On the other hand, poor dietary habits are associated with increased lipid peroxidation and reduced antioxidant capacity, so maternal nutrition can vulnerable oxidative stress in infants. While these studies are observational, consistency across cohorts strengthens the inference that maternal diet quality has intergenerational redox effects.

Environmental exposures act in combination with dietary factors to influence redox homeostasis. Pollutants, endocrine-disrupting chemicals, and lifestyle-related stressors increase oxidative load and may overwhelm the antioxidant systems of placenta. A comprehensive review by Sun et al. [35] identifies oxidative stress as a convergent mechanism that links environmental and dietary factors to GDM. However, exposure assessment remains a major methodological limitation. The review highlights how these identified stress factors can influence redox imbalance and metabolic dysregulation during pregnancy. Maternal diet and environmental exposures play critical roles in shaping redox homeostasis during pregnancy. While epidemiological evidence is strong, causality remains difficult to establish. Integrative studies combining dietary assessment, exposure measurement, and molecular redox profiling are needed.

### 1.4. Research Gap and Scope of the Review

Despite our increasing understanding of redox regulations during pregnancy, a significant research gap persists. This is because molecular mechanisms, nutrition, and environmental exposures are often studied apart rather than together. Most existing research focuses on single pathways, which limits our knowledge of how these factors interact and affect oxidative balance at the maternal–placental–fetal interface. There is a clear need for research that combines molecular redox biology with a detailed assessment of diet and environmental influences. In this context, the MD appears as an attractive dietary pattern, because it is a rich source of antioxidants, polyphenols, unsaturated fatty acids, and anti-inflammatory bioactive compounds, which protects oxidative balance. Importantly, emerging evidence suggests that the redox-related benefits associated with MD adherence arise from the synergistic interaction of multiple dietary components, rather than from isolated nutrients or antioxidant supplementation.

Evidence suggests that adherence to the MD can influence gene expression, mitochondrial function, and redox-sensitive signaling pathways, helping to maintain redox stability during pregnancy. A more comprehensive framework to understand redox regulation can be composed of integrating molecular, nutritional, and environmental perspectives, with a focus on MD. This integrated perspective may enhance the development of dietary strategies aimed at supporting redox balance during pregnancy although they should not be interpreted as evidence of direct prevention of pregnancy complications. Future research should focus on therapeutic approaches using long-term redox profiling, dietary interventions, and integration of mitochondrial and vascular biomarkers.

## 2. Overview of the Mediterranean Diet and Its Bioactive Components

### 2.1. Core Dietary Pattern

The MD is widely recognized as a healthy diet which eliminates the risk of chronic diseases such as cardiometabolic, inflammatory, and neurodegenerative diseases. Evidence supporting these benefits is strongest from large prospective cohorts and randomized trials, although direct extrapolation to pregnancy requires caution. In pregnancy, MD adherence has been primarily evaluated through observational cohort studies rather than randomized interventions, limiting causal inference. The main characteristics of MD are daily consumption of vegetables, whole grains, legumes, fruits, nuts, and extra-virgin olive oil (EVOO), many times a week fermented dairy products, moderate consumption of fish and low intake of red and processed meats. However, MD is a complex nutritional whole-diet model that includes variety of foods and nutrients rather than focusing on a single-nutrient intervention. This conclusion also supports MD’s influence on metabolic and redox-sensitive pathways as a result from the synergistic action of multiple bioactive components [36,37,38].

In MD, the main source of dietary fat is EVOO, which has high content of monounsaturated fatty acids, particularly oleic acid. In addition, EVOO includes a wide variety of phenolic compounds such as hydroxytyrosol, tyrosol, and oleuropein derivatives. Human intervention studies outside pregnancy demonstrate that these phenolics demonstrate antioxidant and anti-inflammatory functions and have been shown to regulate endothelial function, lipid oxidation, and redox signaling [39]. Lately, MD is characterized as a nutrigenomic dietary pattern after recent evidence which highlights that EVOO phenolics can influence gene expression related to oxidative stress and mitochondrial function [40].

MD bioactives are mainly found in plant-based foods. Polyphenols, carotenoids, vitamin C, folate, selenium and other antioxidants, which are found in fruits and vegetables, contribute to the regulation of oxidative balance. Recent cohort and intervention studies suggest that a similar diet to plant-rich MD may reduce biomarkers of oxidative stress and inflammation [41]. The most interesting finding is that whole food rather than single nutrients affects the previous results, highlighting the importance of overall dietary patterns.

Fiber, minerals, and phytochemicals, which support metabolic flexibility and glycemic control, are found in legumes, whole grains, and nuts. Nuts provide polyphenols and unsaturated fatty acids that contribute to antioxidant capacity, while legumes supply slowly digestible carbohydrates that reduce oxidative responses after meal [42]. Fish and seafood, consumed regularly but moderately, are key sources of long-chain omega-3 fatty acids, which influence anti-inflammatory effects and can indirectly stabilize redox homeostasis through membrane and mitochondrial mechanisms [43,44]. The MD is supported by strong evidence in non-pregnant populations and growing observational evidence in pregnancy. Its relevance to redox homeostasis lies in the synergistic effects of multiple bioactive components. However, pregnancy-specific randomized trials remain limited, and claims often rely on indirect or experimental data.

### 2.2. Key Antioxidant and Anti-Inflammatory Constituents

Polyphenols, omega-3 fatty acids, and key antioxidant micronutrients converge on similar pathways that regulate redox homeostasis during pregnancy, a period in which physiological ROS signaling must be balanced against excessive oxidative load to preserve placental function and maternal vascular adaptation [45]. Among polyphenols, olive-derived secoiridoids and phenolic alcohols, particularly oleuropein and hydroxytyrosol, have gained attention because they are highly relevant to MD diet and because they interact directly with placental cells. Recent experimental evidence indicates that oleuropein can influence trophoblast functional behaviors, reinforcing the concept that polyphenols may act not only as “antioxidants” in a narrow sense, but also as regulators of redox-sensitive placental physiology [46]. However, these findings should be interpreted as support for dietary patterns rich in plant-based foods, rather than evidence for the efficacy of isolated compounds or supplements during pregnancy. In parallel, resveratrol has been explored primarily through its anti-inflammatory and redox-modulating properties in pregnancy [47]. However, the clinical evidence base remains limited and recent reviews underline that possible benefits must be balanced against uncertainty about dose, timing, and fetal safety [48,49]. Flavonoids found in vegetables, legumes, and fruits have also been shown in experimental studies to reduce oxidative stress and influence blood vessel function in conditions related to pregnancy hypertension, but more well-designed studies in pregnant populations are still needed [50,51].

Omega-3 fatty acids, such as eicosapentaenoic acid (EPA) and docosahexaenoic acid (DHA), represent another major dietary component with relevance to redox regulation and pregnancy outcomes. EPA and DHA are converted into molecules that reduce inflammation, support blood vessel function, and may help lower oxidative stress at the maternal–placental interface [52]. Beyond their established roles in membrane structure and inflammatory resolution, omega-3 fatty acids influence lipid metabolism and mitochondrial function, processes that are closely linked to the production of oxidative stress. Altered fatty acid metabolism contributes to adverse pregnancy outcomes such as metabolic dysregulation and placental dysfunction [45]. Improvement of vascular function and reduction in inflammatory signaling were observed in pregnant women who had adequate EPA and DHA intake. This may help them to reduce oxidative load and improve blood flow to placenta. Overall, current evidence favors dietary adequacy and pattern-based approaches, complemented by targeted, status-based supplementation where clinically indicated, rather than routine high-dose antioxidants use. Moreover, although dietary patterns and isolated nutrient interventions differ conceptually, many infertility supplements rely on inadequately dosed single nutrients with limited evidence, highlighting the need for standardized, human-centered research distinguishing holistic diets from supplements [53,54]. Systematic reviews and meta-analyses report that omega-3 supplementation notably helps to reduce risk of preterm and early preterm birth, although this cannot be applied to other maternal and neonatal outcomes [55,56]. Recent international clinical practice guidelines further support these findings by recommending adequate omega-3 fatty acid intake during pregnancy, especially DHA, as a preventive strategy for preterm birth, with the strongest benefits observed in women with low baseline omega-3 status [57].

**Figure 1:**
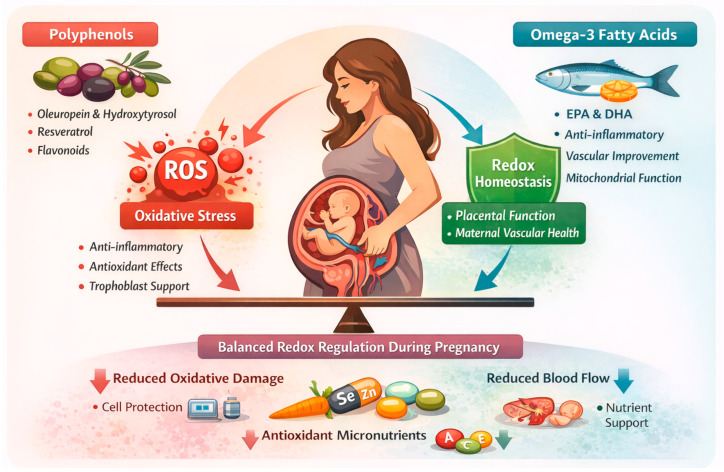
Possible connection between key antioxidants and anti-inflammatory constituents, including polyphenols and omega-3 fatty acids with balanced redox regulation during pregnancy (created by the authors using Adobe Photoshop 2026 (Version 27.0)). ROS: reactive oxygen species; EPA: eicosapentaenoic acid; DHA: docosahexaenoic acid; Se: Selenium; Zn: Zinc; A: Vitamin A; C: Vitamin C; E: Vitamin E.

Another important group of antioxidants includes micronutrients particularly vitamins C and E, selenium, zinc, and carotenoids, which help control oxidative balance through different actions, including neutralizing free radicals, protecting cell membranes, and supporting antioxidant enzymes. Pregnancy cohort evidence has linked low first-trimester vitamin E status with higher preeclampsia risk, underlying that correcting specific deficiencies is different from using high-dose supplements in all cases [58]. Selenium is important because it supports antioxidant enzymes, and reviews of pregnancy hypertension show that lower selenium levels are linked to disease risk, although it remains unclear whether low selenium is a cause, a marker, or a consequence of the disease process [59]. Recent reviews suggest that selenium may improve antioxidant defenses in women with low levels. Evidence for a causal role in preventing pregnancy complications remains limited, favoring targeted, status-based supplementation over routine high-dose use in all pregnancies [32]. Carotenoids such as β-carotene and lutein are also important, as studies show that higher carotenoid levels are linked to better maternal blood vessel function in late pregnancy and after birth, suggesting a role in vascular and oxidative balance [60]. Dietary intake studies have examined relationships between carotenoid intake patterns and preeclampsia risk, although results vary by situation and should be interpreted with caution because of other influencing factors and limits in dietary measurement [61]. Among MD components, omega-3 fatty acids have the strongest clinical evidence for pregnancy benefit, while polyphenols and micronutrients are supported mainly by observational data. Consequently, evidence favors dietary adequacy and targeted supplementation over indiscriminate high-dose antioxidant use. Overall, pregnancy-focused recent evidence supports that these antioxidant nutrients are more effective when they are obtained from high-quality diets or targeted supplements based on individual needs and nutrient status, rather than from general high-dose antioxidant use across all pregnancies [9,57,62]. Possible connection between key antioxidants and anti-inflammatory constituents with redox regulation during pregnancy is shown in Figure 1.

Despite the well-documented antioxidant and anti-inflammatory benefits of MD analyzed earlier, there is growing evidence that these protective effects of MD may be influenced by concurrent exposure to environmental contaminants present in commonly consumed foods, such as fish, seafood, fruits, vegetables, and cereals. Previous studies in pregnant women showed that adherence to the MD is linked with increased oxidative stress biomarkers linked to heavy metals like cadmium, nickel, lead, and mercury, along with essential elements such as selenium and zinc [63]. Similarly, adherence to MD has been connected to increased exposure to POPs, including polychlorinated biphenyls and per- and polyfluoroalkyl substances, which may affect MD’s protective effects. Recent research suggested that pregnant women who followed MD along with low POP exposure had a lowest risk of gestational diabetes mellitus, showing that benefits from MD can be influenced by chemical contaminants [64,65]. These findings underscore the need to integrate nutritional guidance with environmental health considerations. MD remains a scientifically grounded model that aligns human health promotion with environmental sustainability [66], while climate change, food system disruptions, and regulatory gaps increasingly threaten food quality and safety, disproportionately affecting women during pregnancy [67].

### 2.3. Bioavailability and Maternal–Fetal Transfer

Maternal adherence to the MD during pregnancy has important implications for placental nutrient handling and fetal exposure to bioactive compounds, shaping the intrauterine environment through metabolic, inflammatory, and redox-sensitive pathways. The placenta is not a passive barrier but a highly active metabolic organ that senses maternal dietary signals and regulates the transfer of nutrients, lipids, and micronutrients to the fetus. Dietary patterns characterized by low inflammatory potential, such as the MD, are increasingly associated with favorable placental function and improved perinatal outcomes. The importance of maternal diet quality for placental and fetal health is certified by data from the IMPACT BCN trial, which demonstrates that a lower maternal Dietary Inflammatory Index during pregnancy which reflects a Mediterranean-style diet is linked to better perinatal outcomes [68].

Systematic reviews have demonstrated how the placenta transfers nutrients efficiently and how effectively its vascular network functions, examining dietary patterns during pregnancy and MD adherence which is linked to lower circulating inflammatory markers and reduced oxidative stress [69,70]. Moreover, the placenta is a central interface where maternal diet can influence oxidative balance and nutrient delivery to the fetus. For this reason, oxidative stress markers in maternal and placental tissues are strongly linked to pregnancy outcomes [70]. Oxidative stress in the placenta may decrease with the intake of antioxidants, unsaturated fatty acids, and micronutrients from the MD, which can help ensure more stable nutrient transfer to the fetus. However, these studies rely primarily on biomarkers rather than direct placental measurements, limiting direct analysis.

Recent evidence also reports that placental structure and molecular regulation is affected by the quality of maternal diet. Lecorguillé et al. [71] found associations between maternal dietary inflammation and changes including placental development, patterns of fetal growth, and DNA methylation. These findings suggest that MD components have the potential to cause epigenetic changes in the placenta and can modulate placental oxidative stress pathways and inflammatory signaling. This conclusion is inconsistent to Westernized diet components that promote redox imbalance and placental dysfunction [72].

Beyond nutrient transfer, the placenta may take up bioactive compounds from the MD that influence fetal development through epigenetic, and pathways linked to the gut microbiota. Recent reviews highlight that maternal nutrients and metabolites involving gut bacteria interact with the placental epigenome, influence gene expression related to growth, metabolism, and oxidative stress regulation in the fetus [73]. While this concept is supported by experimental and integrative human studies, causal pathways remain speculative. Overall, the link between adherence to the MD, better fetal growth, and long-term health outcomes is explained by this integrated placental response. Maternal MD adherence is consistently associated with favorable placental and perinatal outcomes, but evidence in humans is still emerging. Epigenetic and microbiota-mediated pathways are promising but require further validation.

## 3. Oxidative Stress and Redox Homeostasis in Pregnancy

### 3.1. Sources of Reactive Oxygen and Nitrogen Species (ROS/RNS)

During pregnancy, there is an increased metabolic demand, and redox homeostasis is strictly regulated. This mechanism is required to support placental development, maternal adaptation and fetal growth. It is already known that oxidative stress occurs when the balance between ROS generation and antioxidant defenses is disrupted, while recent studies support that when redox balance is impaired, there is higher risk of a wide spectrum of pregnancy complications appearing, including preeclampsia, FGR, recurrent pregnancy loss, and adverse neurodevelopmental outcomes [8,32]. When mitochondrial quality control is impaired, issues such as defects in biogenesis, an imbalance in fusion and fission processes, and mitophagy can lead to excessive ROS production that overwhelms antioxidant defenses [74,75]. Evidence for these mechanisms is strongest in experimental models, with supportive but largely associative data from human placental studies.

Placental mitochondria exhibit remarkable plasticity during pregnancy, adapting their ETC activity and use of substrate to change oxygen and nutrient levels. Reductions in oxidative phosphorylation help control ROS production while maintaining ATP synthesis. This is observed particularly in hypoxic environments like early gestation or high altitudes pregnancies [76]. Redox imbalance and reduced placental efficiency appear due to mitochondrial dysfunction, when previous adaptations fail. These changes disrupt energy metabolism, increase oxidative stress, and impair placental vascularization and nutrient transport.

At the cellular level, villous trophoblasts form a key redox-sensitive interface between the maternal and fetal compartments. To maintain placental structure and function, strict control of oxidative stress within trophoblasts is essential. This happens due to excessive ROS promoting DNA damage, apoptosis, and premature cellular senescence. DCTPP1 is a nucleotide metabolism enzyme, very crucial for maintaining oxidative stress balance in human trophoblasts [77]. This enzyme controls redox-related gene expression after transcription, illustrating the complexity of intracellular antioxidant control mechanisms [78]. Placental hormone production is altered when redox balance in trophoblasts is disrupted. This trophoblast redox balance disruption also impairs spiral artery remodeling and restricts fetal growth. These pathological features are commonly observed in cases of placental dysfunction and are strongly associated with FGR and severe fetal outcomes [14,79].

Maternal physiology and long-term offspring health is influenced by oxidative stress that extends beyond placenta. Follow-up cohort studies demonstrate that redox imbalance during critical developmental periods, like pregnancy, may have long-term consequences, such as early childhood neurodevelopment which is linked with maternal oxidative stress biomarkers [80]. As some comprehensive reviews have demonstrated trimester-specific changes are observed in lipid peroxidation products, antioxidant enzyme activity, and redox-sensitive metabolites, confirming that oxidative balance is not static across gestation [9,81].

Furthermore, oxidative stress influences inflammation and immune modulation at the maternal–fetal interface. ROS activates innate immune signaling pathways, which leads to an increase in cytokine production and promotes endothelial dysfunction. In pathological pregnancies, such as preeclampsia and HELLP syndrome, sustained oxidative stress and activation of the innate immune reinforced each other, maintaining chronic inflammation [82,83]. Additionally, modified nutrient metabolism raises mitochondrial ROS levels and inflammatory signaling in trophoblasts and immune cells [16]. Single-cell analyses found changes in redox status and immune dysregulation over time, indicating that immune and trophoblast subpopulations vary between early and late stages of preeclampsia.

Overall, current evidence indicates that oxidative stress is not only a result of pregnancy pathology but a key factor linking mitochondrial dysfunction, trophoblast maladaptation, and immune activation. Experimental data clarify mechanisms, while human evidence strongly supports association with adverse outcomes. Key gaps remain in defining causal timing and thresholds for intervention. Once again, understanding these mechanisms around redox homeostasis during pregnancy is crucial for identifying risk biomarkers and developing targeted interventions.

### 3.2. Antioxidant Defense Systems

Pregnancy involves significant metabolic and vascular changes that increase ROS production in maternal and placental tissues. Usually, an antioxidant defense system which maintains redox balance supports placental development, vascular adaptation, and fetal growth controls oxidative stress. Pregnancy complications like preeclampsia, placental insufficiency, and adverse neonatal outcomes appear due to excessive oxidative stress when this balance is disturbed [8,84,85]. There are two antioxidant defenses in pregnancy, with enzymatic and non-enzymatic components, which combine to neutralize ROS and limit cellular damage.

Among enzymatic antioxidants, superoxide dismutase (SOD), glutathione peroxidase (GPx), and catalase play central roles in placental and systemic redox control. SOD is the primary defense against oxidative stress which converts superoxide radicals into hydrogen peroxide. This hydrogen peroxide is then detoxified by catalase and GPx. These enzymes are found in placental trophoblasts, endothelial cells, and maternal circulation, highlighting their importance at the maternal–fetal interface [10,85]. Reduced SOD and GPx activity are often accompanied by increased lipid peroxidation and endothelial dysfunction, in hypertensive disorders of pregnancy and preeclampsia, where modified activity of the previous antioxidant enzymes has been observed [86,87]. Zinc is an essential cofactor for antioxidant enzymes, and when its levels are reduced, that leads to reduced enzymatic activity and adverse neonatal outcomes in preeclampsia [88]. These findings support that impaired enzymatic antioxidant defenses contribute to oxidative injury in complicated pregnancies.

An extra level of protection is offered by non-enzymatic antioxidants, which directly scavenge ROS and support enzymatic systems. Among these, glutathione is the most commonly found intracellular antioxidant and maintains redox balance in placental cells. It plays a key role in detoxifying peroxides, because it acts as a substrate for GPx and preserves cellular integrity. It is very important during pregnancy because when there is reduced glutathione availability, placental insufficiency and adverse fetal outcomes arise [78,89]. Antioxidant vitamins, particularly vitamins A, C, and E, neutralize free radicals and protect lipids and membranes from oxidative damage, contributing to redox homeostasis. Limited concentrations of these vitamins in circulation of blood and in placenta have been observed in preeclampsia, indicating insufficient antioxidant capacity in affected pregnancies [90,91].

Another category of non-enzymatic antioxidants with anti-inflammatory and redox-modulating properties is diet-derived polyphenols. MD, which is a rich source of polyphenols, has been associated with increased oxidative balance and reduced inflammation during pregnancy [65,92]. Systematic reviews suggest that polyphenols may support antioxidant defenses and endothelial function in preeclampsia, although clinical evidence remains heterogeneous [93]. Moreover, astaxanthin is another compound that has shown antioxidant and anti-inflammatory effects in reproductive disorders and assisted reproduction. This further supports the therapeutic potential of non-enzymatic antioxidants in redox-related pregnancy conditions [94,95].

In conclusion, the combined action of enzymatic and non-enzymatic components offers antioxidant defense systems in pregnancy, which maintains redox balance. When these systems are disrupted, placental function impairs and vulnerability to oxidative stress-related complications is increased. The definition of these antioxidant defenses’ function and impairment during pregnancy is essential for improving risk assessment and developing targeted nutritional and therapeutic strategies. While deficiency states clearly increase risk, broad antioxidant supplementation has not proven effective. Targeted, diet-based strategies aligned with individual redox status appear most promising.

## 4. Mediterranean Diet as a Countermeasure to Pollutant-Induced Oxidative Stress During Pregnancy

Exposure to environmental pollutants, including airborne particulate matter (PM), persistent organic pollutants (POPs), heavy metals, and pesticides, during pregnancy increase risks to health of both the mother and the fetus, with oxidative stress being a key mediating factor [96]. Environmental pollutants are involved in ROS generation, leading to oxidative stress, and damaging proteins, lipids and DNA, as well as disrupting the cellular signaling pathways. In fact, exposure of pregnant women to polycyclic aromatic hydrocarbons (PAHs) has been associated with increased oxidative stress in both pregnant women and neonates, demonstrating in utero oxidative injury due to high pollution [97]. However, it is interesting to highlight that when several pollutants are combined, like endocrine-disrupting chemicals and heavy metals, synergistic effects of PM mixtures amplify oxidative stress and developmental toxicity [98] or inflammatory signaling [99,100]. But what about air pollution and its association with oxidative stress biomarkers? Previous research suggests that maternal exposure to air pollutants can ultimately affect biomarkers oxidative stress (Table 1).

Keeping in mind that pollution affects pregnancy complications implied through oxidative stress, nutritional strategies that enhance antioxidant defense have gained increasing interest. MD is especially considered as a particularly promising dietary pattern through its antioxidants and anti-inflammatory properties, since it is characterized by high consumption of fruits, vegetables, legumes, nuts, whole grains, and EVOO, moderate intake of fish rich in omega-3 polyunsaturated fatty acids (PUFAs), and limited intake of red and processed meats [112].

**Figure 2:**
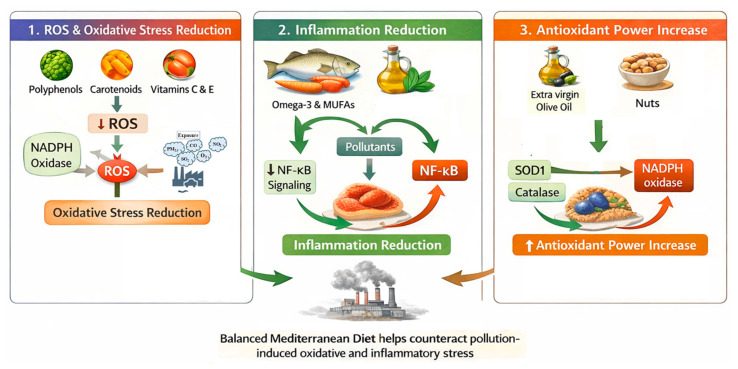
Protective role of MD against oxidative stress related to pollution (created by the authors using Adobe Photoshop 2026 (Version 27.0)). NADPH oxidase: nicotinamide adenine dinucleotide phosphate oxidase; ROS: reactive oxygen species; PM: fine particulate matter; CO: carbon oxide; NO_2_: nitric dioxide; SO_2_: Sulfur dioxide; O_3_: ozone; MUFAs: monounsaturated fatty acids; NF-κB: nuclear factor kappa-light-chain-enhancer of activated B cells; SOD1: superoxide dismutase 1.

MD has been associated with lower oxidative stress biomarkers, a finding commonly attributed to its content of polyphenols, carotenoids, flavonoids, and antioxidant vitamins (C and E), which can directly scavenge reactive oxygen species and influence mitochondrial pathways involved in ROS generation and redox signaling; experimental and observational studies suggest that specific polyphenols such as hydroxytyrosol, oleuropein, and quercetin may downregulate NADPH oxidase activity, reduce downstream ROS production, and modulate AMPK- and NF-κB–dependent inflammatory pathways, effects that are biologically plausible in the context of pollutant-induced oxidative stress, although evidence for a direct protective effect of MD against pollution-related oxidative injury during pregnancy remains largely indirect and not yet supported by pregnancy-specific interventional trials [113,114]. Oxidative stress and inflammation are interwoven, with pollutant exposure triggering pro-inflammatory transcription factors such as NF-κB, which in turn amplifies ROS production. Monounsaturated fatty acids (MUFAs) from olive oil and omega-3 PUFAs modulate inflammation and reduce inflammatory biomarkers (C-reactive protein and interleukin-6), indicating an anti-inflammatory factor that directly counters pollutant-induced oxidative cascades [115]. Moreover, polyphenols may inhibit NF-κB activation and related cytokine release and therefore decrease both inflammation and ROS generation. [113]. Additionally, long-term adaptation of MD increases organism’s antioxidant power [112], with EVOO or nuts exhibiting particularly higher plasma SOD and catalase activity compared with control diets, and lower pro-oxidant xanthine oxidase activity [116]. Also, MD components can influence gene expression of key oxidant/antioxidant regulators such as SOD1 and catalase, while reducing expression of pro-inflammatory NF-κB subunits [117] (Figure 2).

During pregnancy, oxidative stress is known to be counteracted by MD, which counteracts endothelial dysfunction, a common feature of both air pollution toxicity and pregnancy disorders, through EVOO phenolics that enhance nitric oxide bioavailability [34]. Moreover, MD is associated with improved insulin sensitivity and lipid metabolism, which further reduce ROS generation, providing indirect protection against oxidative stress [112], while at the same time, MD is linked to lower psychosocial stress and improved well-being in pregnant women, highlighting interconnected pathways linking stress, inflammation, and redox balance [118]. Adherence to the MD has been associated with a lower incidence of certain pregnancy complications and markers of improved placental function, suggesting a potentially more favorable intrauterine environment that may be more resilient to oxidative stress, including that induced by environmental pollutants [119]. More broadly, omega-3 fatty acids and vitamins E and B have been shown to modulate pathways related to particulate matter–induced inflammation, oxidative stress, epigenetic alterations, and cardiovascular injury in experimental models and non-pregnant human studies; however, direct evidence of protective efficacy in pregnant populations remains limited [100]. Maternal nutrition, particularly rich in antioxidants such as MD, plays a crucial role in early developmental processes such as implantation, placentation, and organogenesis, with significant potential to improve pregnancy outcomes and reduce oxidative stress related pregnancy disorders. In this context, intervention strategies with health-promoting nutrition, including antioxidant therapies, may offer effective primary prevention against adverse developmental outcomes associated with maternal exposure to environmental pollutants. Climate change, food insecurity, and fluctuating environmental pollution could influence prenatal and postnatal health, which is why coordinated international research and preventive strategies supported by public education should be introduced [98].

## 5. Conclusions

The current review highlights oxidative stress as a key factor between environmental pollutant exposure and adverse pregnancy outcomes. MD characterized by antioxidant and anti-inflammatory-rich properties offers an effective nutritional strategy enhancing maternal redox resilience, improving metabolic and vascular function, and possibly mitigating pollutant-induced molecular damage. Nevertheless, potential interference of certain environmental contaminants in common MD foods undermines the protective role of MD and underscores the need for integrated nutritional and environmental health strategies. Consequently, nutrition cannot be considered in isolation, calling for a more holistic approach to maternal health. Dietary recommendations alone are insufficient to protect maternal–fetal health in polluted environments. That is why effective prevention requires coordinated nutritional guidance, environmental exposure reduction, and food safety regulation to preserve the biological benefits of healthy diets. In this context, future randomized trials, research studies, and policy interventions are required to address these gaps by clarifying causality, refining dietary recommendations, and reducing chemical exposures, aimed at highlighting the effects of MD and safeguarding maternal and fetal health in polluted environments.

## Figures and Tables

**Table 1:** Maternal exposure to air pollutants and their association with oxidative stress biomarkers. 8-OHdG: 8-hydroxy-2′-deoxyguanosine; PFAS: per- and polyfluoroalkyl substances; PFOS: perfluorooctanesulfonic acid; PM: fine particulate matter; NOx: nitric oxide; O_3_: ozone; PAHs: polycyclic aromatic hydrocarbons; NO_2_: nitric dioxide.

Authors, Year	Study Type	Pollutants	Main Results
Zhang et al., 2021 [101]	Cross-sectional	Phthalates	Phthalate metabolites associated with higher 8-OHdG
Taibl et al., 2022 [102]	Cohort	PFAS	PFOS linked to increased oxidative-stress biomarkers (8-isoprostane-prostaglandin-F_2α_)
Aguilera et al., 2023 [103]	Review	PM, NOx, O_3_, PAHs	Oxidative stress plays a role in the connection between prenatal air pollution and adverse birth outcomes
Eick et al., 2023 [104]	Pooled cohort analysis,	Air pollution	Urinary oxidative stress biomarkers (8-iso-prostaglandin-F2α, F2-IsoP-M, and prostaglandin-F2α) associated with air pollutant exposure and preterm birth
Siwakoti et al., 2024 [105]	Review	PFAS	PFOS associated with 8-isoprostanes; sex-specific effects noted on PFAS with 8-OHdG
Wang et al., 2024 [106]	Cohort	PM_2.5_, NO_2_, PAHs	Systemic oxidative stress biomarkers correlated with environmental pollution and pregnancy complications
Almeida-Toledano et al., 2024 [107]	Systematic review	Phthalates	Association between prenatal phthalate exposure and pregnancy complications; oxidative stress a key point
Pizent et al., 2025 [108]	Review	Air pollution, metals, tobacco	Pollutant exposures cause metabolomic oxidative stress alterations
Sun et al., 2025 [109]	Systematic review	Heavy metals	Lead, cadmium, and mercury associated with maternal oxidative stress and impaired fetal growth.
McNell et al., 2025 [110]	Analysis	Phthalates and replacements	Certain phthalates and replacements during pregnancy were connected with implications for oxidative stress and hypertensive disorders
Kou et al., 2025 [111]	Multi-statistical study	Heavy metals	Prenatal heavy metal exposure associated with adverse neurodevelopment, mediated partly by oxidative stress

## Data Availability

No new data were created or analyzed in this study. Data sharing is not applicable to this article.
